# The similarities between smDCs and regDCs in alleviating the immune injury caused by transplantation of hepatocytes differentiated from ESCs

**DOI:** 10.1186/s13287-017-0712-1

**Published:** 2017-11-21

**Authors:** Cheng Zhang, Wenwei Liao, Bing Cai, Furong Liu, Qiong Ke, Xiaofeng Zhu, Xiaoshun He, Anbin Hu

**Affiliations:** 1 Organ Transplant Center of the First Affiliated Hospital, Sun Yat-sen University, Guangdong Provincial Key Laboratory of Organ Donation and Transplant Immunology,Guangdong Provincial International Cooperation Base of Science and Technology(Organ Transplantation), Guangzhou, China; 2 Reproductive Medicine Center of the First Affiliated Hospital, Sun Yat-sen University, Guangzhou, China; 3 Cardiac Surgery Itensive Care Unit of the First Affiliated Hospital, Sun Yat-sen University, Guangzhou, China; 4 Zhongshan Medical School of Sun Yat-sen University, Guangzhou, China

**Keywords:** Embryonic stem cells, Hepatocyte transplantation, Semimature dendritic cells, Regulatory dendritic cells, Regulatory T cells, Low immunogenicity

## Abstract

**Background:**

This study aimed to investigate the tolerogenic mechanisms induced by semimature dendritic cells (smDCs) and regulatory dendritic cells (regDCs) after transplantation of hepatocytes differentiated from mouse embryonic stem cells (ESCs) and to confirm the low immunogenicity of hepatocytes differentiated from ESCs.

**Methods:**

Green fluorescent protein-labeled ESCs collected from 129 mice were cultured to differentiate into hepatocytes. smDCs and regDCs were cultured in vitro. The hepatocytes were cultured after being extracted from the livers of 129 mice. After injecting smDCs or regDCs 3 days in advance, these differentiated hepatocytes and normal hepatocytes were transplanted into the livers of BALB/c mice separately. Subsequently, the histopathological features and cytokines in transplant tissues as well as the Foxp3 expression in peripheral blood CD4^+^ T cells of the recipients were examined.

**Results:**

The morphological phenotypes of smDCs and regDCs were similar. They both expressed medium levels of MHC-II, CD40, CD80, and CD86, high levels of TGF-β and IL-10, and low levels of IL-2. The survival of differentiated hepatocytes was prolonged and inflammatory infiltration in transplant tissues was reduced in both the smDC and regDC groups. Foxp3 expression in peripheral blood CD4^+^ T cells of the smDC group increased to 5.38% and that of the regDC group also rose to 3.87%. Moreover, the inflammatory infiltration in the tissues receiving transplanted hepatocytes was more obvious.

**Conclusions:**

smDCs and regDCs were similar tolerogenic dendritic cells. They both could alleviate the immune injury by inducing CD4^+^CD25^+^Foxp3^+^ regulatory T cells through the medium expression of MHC-II, CD40, CD80, and CD86 and the appropriate secretion of cytokines. Hepatocytes differentiated from ESCs displayed low immunogenicity.

## Background

In recent years, hepatocytes differentiated from embryonic stem cells (ESCs) have achieved excellent results in replacement therapy for hepatic failure or in the transient treatment given prior to liver transplantation [1, 2]. Although ESCs and differentiated cells derived from ESCs show low immunogenicity [3], immune rejection remains a common problem after transplanting hepatocytes differentiated from ESCs [4]. Furthermore, cells differentiated from induced pluripotent stem cells (iPSCs) also trigger immune rejection after transplantation [5]. Therefore, immune injury caused by rejection remains an urgent problem to be solved and limits further clinical application of stem cell transplantation. In our previous research, the differentiation of mouse ESCs into hepatocytes and the transition of mouse bone marrow mononuclear cells into semimature dendritic cells (smDCs) were successfully induced. It has been confirmed that smDCs can induce immune tolerance after mouse skin transplantation and can significantly alleviate immune injury after transplanting hepatocytes differentiated from mouse ESCs [6, 7]. Moreover, as a type of tolerogenic dendritic cells, regulatory dendritic cells (regDCs) play important roles in inducing immune tolerance after stem cell transplantation [8]. However, it remains unclear whether smDCs are similar to regDCs in terms of cell morphology, immunological phenotype, and their mechanisms of alleviating immune injury after transplanting hepatocytes differentiated from mouse ESCs. This study aimed to identify the links between smDCs and regDCs in inducing tolerance after transplanting hepatocytes differentiated from mouse ESCs. The findings of this study are beneficial to promote the clinical applications of stem cell transplantation and to provide some insights for transplantation tolerance.

## Methods

### Animals and ESCs

Specific pathogen-free 129 mice and BALB/c mice (age range 6–8 weeks) were obtained from the Experiment Animal Center of Sun Yat-sen University in Guangzhou, China. The experimental protocol was approved by the Sun Yat-sen University Animal Ethics Committee. Green fluorescent protein (GFP)-labeled mouse ESCs from 129 mice and ESCs lacking fluorescence were donated by Professor Huang Bing at the Zhongshan Ophthalmic Center of Sun Yat-sen University.

### Differentiation of ESCs into hepatocytes and establishment of hepatocyte transplantation models

ESC culture reagents and detection reagents were: DMEM (Gibco BRL), 20% fetal bovine serum (SJQ, Hangzhou, China), 0.1 mol/L beta-mercaptoethanol (Gibco BRL), 25 mmol/L HEPES (Gibco BRL), 2 mmol/L l-glutamine (Gibco, USA), 100 U/ml penicillin, 100 μg/L streptomycin (Gibco BRL), 1000 U/ml recombinant mouse leukemia inhibitory factor (LIF) (Gibco BRL), 100 ng/ml recombinant mouse acidic fibroblast growth factor (aFGF), 20 ng/ml recombinant mouse hepatocyte growth factor (HGF), and 10 ng/ml recombinant mouse oncostatin M (OSM) (Sigma). Rabbit anti-murine alpha-fetoprotein (α-AFP) antibody (DAKO, Denmark), rabbit anti-murine albumin antibody (Biodesign, USA), and goat anti-rabbit IgG/FITC/TMRITC were used.

To track the markers, ESCs with fluorescence were injected into the mouse liver. In order to avoid confusion, we used ESCs without fluorescence in the in-vitro differentiation phase. ESCs without fluorescence were used when detecting the expression of ALB. The GFP-labeled ESCs were cultured and differentiated into hepatocytes, which could reach a purity of nearly 70% after cell sorting. The details of cell sorting could be seen in our previous publication [9]. In brief, to induce differentiation, ESCs were suspended in DMEM containing 4.5 mg/ml glucose, 20% fetal bovine serum, 2 mM l-glutamine, 25 mM HEPES, 100 mg/ml penicillin, 100 mg/ml streptomycin, and 0.1 beta-mercaptoethanol, without recombinant mouse LIF. ESCs were incubated by a suspension culture method (2 × 10^4^ cells/1 ml medium) for 5 days to develop into embryoid bodies (EBs). The resulting EBs were then plated onto tissue culture plates coated with 0.1% gelatin and allowed to attach and spread in further culture with differentiation medium. Several growth factors were added into culture medium for hepatocyte differentiation, including 20 ng/ml transforming growth factor (TGF), 20 ng/ml recombinant human AFP, 100 ng/ml recombinant mouse aFGF, 20 ng/ml recombinant mouse HGF, 10 ng/ml recombinant mouse OSM, 10^–7^ M dexamethasone (DEX), 5 mg/ml insulin, 5 mg/ml transferrin, and 5 mg/ml selenious acid (Gibco BRL). These growth factors were added to the medium at the following times: as early stage factors, aFGF was added to EBs on days 7–11, and TGF and AFP were added on days 7–19; as middle stage factors, HGF was added on days 11–19; and as late stage factors, OSM, DEX, insulin, transferrin, and selenious acid were added on days 15–19.

The intracellular expression of AFP and ALB was detected by radioimmunoassay (RIA) on the 15th day of ESC differentiation in vitro. Moreover, hepatocytes of 129 mice were isolated from their livers. During hepatocyte isolation, the liver was cut into pieces using a pair of tissue scissors and washed with PBS three times. Then 0.5% collagenase was used to break the liver samples into a single cell suspension, which was treated with an erythrocyte lysis buffer to remove red blood cells. Hepatocytes were harvested 3 days after culture in a DMEM medium containing 20% fetal bovine serum (SJQ), 2 mmol/L l-glutamine, 25 mmol/L HEPES, 100 U/ml penicillin, and 100 μg/L streptomycin. Subsequently, the suspensions containing ESCs or regular hepatocytes were respectively infused into liver parenchyma of different BALB/c mice using a 100-μl microinjector, to establish a model of hepatocyte transplantation.

### Inducing and harvesting smDCs and regDCs

Culture reagents were RPMI 1640 (Gibco, USA), 10% fetal bovine serum (SJQ), 100 U/ml penicillin, 100 μg/L streptomycin (Gibco BRL), 2 mmol/L l-glutamine (Gibco, USA), 20 μg/L recombinant mouse granulocyte–macrophage colony-stimulating factor (rmGM-CSF), 20 μg/L recombinant mouse interleukin-4 (rmIL-4), 40 ng/ml tumor necrosis factor alpha (TNF-α), 20 μg/L interleukin-10 (IL-10), 20 μg/L human TGF-β1 (PeproTech, USA), and 1 μg/ml lipopolysaccharide (LPS) (Sigma, USA).

According to our previous methods [10], bone marrow mononuclear cells could be extracted from the femurs and tibia of mice. Bone marrow mononuclear cells were cultured in a RPMI-1640 medium containing 10% fetal bovine serum, rmGM-CSF (20 μg/L), rmIL-4 (20 μg/L), 100 U/ml penicillin, 100 μg/L streptomycin, and 2 mmol/L l-glutamine for 9 days to differentiate into dendritic cells, which were cultured with TNF-α (40 ng/ml) for 24 h to generate smDCs. The immature dendritic cell (imDC) is a state before the smDC. In addition, bone marrow mononuclear cells were cultured with LPS (1 μg/ml) for 24 h to generate mature DCs (mDCs).

As described in [11, 12], the bone marrow mononuclear cells harvested from femurs and tibia of 129 mice were cultured with a RPMI-1640 medium containing 10% fetal bovine serum, rmGM-CSF (20 μg/L), IL-10 (20 ng/ml), human TGF-β1 (20 ng/ml),100 U/ml penicillin, 100 μg/L streptomycin, and 2 mmol/L l-glutamine for 8 days, followed by LPS (1 μg/ml) treatment of 24 h to generate regDCs.

### Induced immune tolerance in the model of hepatocyte transplantation

The subjects were divided into the following groups: the smDC group, the imDC group, the mDC group, the regDC group, and the PBS group. There were 18 mice in each group. Firstly, each BALB/c mouse was infused with 0.5 ml of smDC suspension (2 × 10^5^/mouse) through the tail vein. Three days later, these 18 mice were further divided into two groups. Using a 100-μl microinjector, the suspension of ESC-derived hepatocytes was infused into the liver parenchyma of BALB/c mice in the first group. Similarly, the suspension of regular hepatocytes was infused into the liver parenchyma of BALB/c mice in the other groups. In the other four treatment groups, similar experimental procedures were performed.

### Observation of cell morphology

A fully automated and inverted microscope (Zeiss, Germany) was used to observe the differentiation of ESCs into hepatocytes. The morphological features of smDCs, mDCs, imDCs, and regDCs were also observed.

### Flow cytometry analysis and detection of secreted cytokines

Relevant reagents were PE-labeled CD86 antibody, BB515-labeled major histocompatibility complex II (MHC-II) (I-A^b^) antibody, PerCP-Cy5.5-labeled CD80 antibody, and APC-labeled CD40 antibody. An IL-2 reagent kit, IL-10 reagent kit, and TGF-β reagent kit were used.

The expression of MHC-II, CD40, CD80, and CD86 on the surface of smDCs, mDCs, imDCs, and regDCs was determined by flow cytometer. Cytokines such as IL-2, IL-10, and TGF-β were secreted by smDCs, mDCs, imDCs, and regDCs, and were detected using an enzyme-linked immunosorbent assay.

To demonstrate the effective regulatory activity of smDCs and regDCs, peripheral blood CD4^+^ T cells from the recipient mice were cultured with smDCs, regDCs, imDCs, and mDCs from 129 mice or PBS in vitro for 24 h, respectively. The expression of CD4^+^CD25^+^FoxP3^+^ T cells was detected by flow cytometry.

### Observation of the growth of transplanted cells and detection of immunological indexes

In each group, two mice (one mouse from each of the two subgroups) were randomly selected to observe their quality of life and survival. Subsequently, four mice were randomly chosen from each experimental group and sacrificed (two mice from each of the two subgroups) to acquire liver tissues and peripheral blood during the 1st, 2nd, 3rd, and 4th week after transplantation, respectively. The liver tissues were cut into liver sections and the infiltration of CD3^+^ T lymphocytes into the transplant tissues was detected using immunohistochemistry assays. An automatic fluorescence microscope was used to observe the growth of transplanted cells and the infiltration of CD3^+^ T lymphocytes. Meanwhile, the expression of FoxP3 in peripheral blood CD4^+^ T cells was detected with a flow cytometer. The relevant antibodies were rabbit anti-murine CD3 antibody (DAKO, Denmark), PE-labeled FoxP3 antibody, and FITC-labeled CD4 antibody (both Becton Dickinson Company, USA).

### Statistical analysis

All data are presented as mean ± SD. Data between different groups were compared with ANOVA followed by *t* test, where *P* < 0.05 was considered significant.

## Results

### ESCs differentiated into hepatocytes

**Fig. 1:**
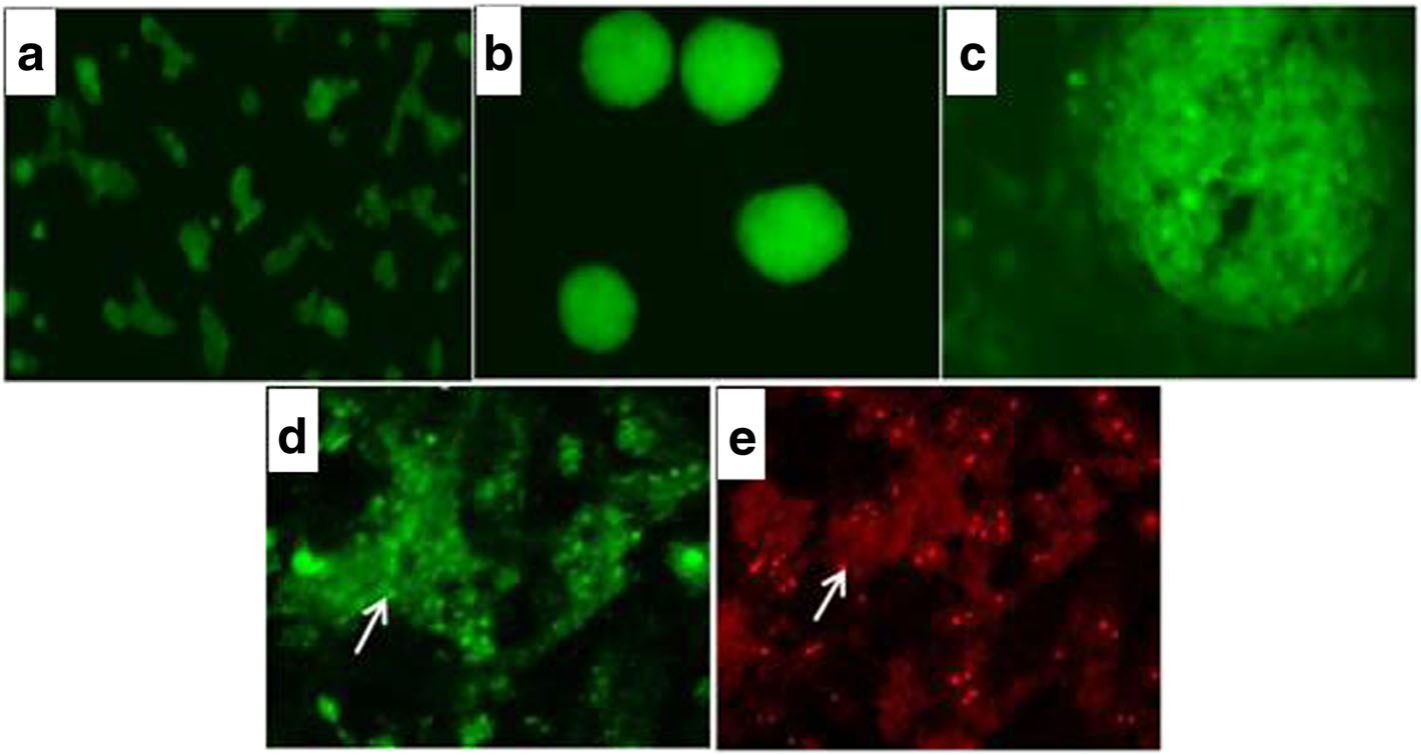
GFP-labeled ESCs from 129 mice differentiated into hepatocytes. **a** ESCs. **b** EBs. **c** EBs differentiated into hepatocytes. In the late stage of differentiation, there were polygonal hepatocyte-like cells 20–30 μm in diameter at the center and edge of the EB cell colony. These cells had large and round nuclei. **d** Intracellular expression of ALB detected by RIA on the 15th day of ESC differentiation in vitro (*arrow*). Parts of day 15 EBs cultured with growth factors were stained with antibodies against ALB (FITC). **e** Intracellular expression of AFP was also detected by RIA on the 15th day of ESC differentiation in vitro (*arrow*). Parts of day 15 EBs cultured with growth factors were stained with antibodies against AFP (TMRITC)

Using the automatic and inverted fluorescence microscope, it could be seen that ESCs differentiated into hepatocytes through the stage of embryoid bodies (EB). In the late stage of differentiation, it was found that there were polygonal hepatocyte-like cells 20–30 μm in diameter at the center and edge of the EB cell colony. These cells had large and round nuclei. In RIA detection assays, the specific markers of hepatocytes such as AFP and ALB were observed in these hepatocyte-like cells differentiated from ESCs (Fig. 1a–e).

### Cell morphology, immunological phenotype. and cytokine secretion of smDCs and regDCs

**Fig. 2:**
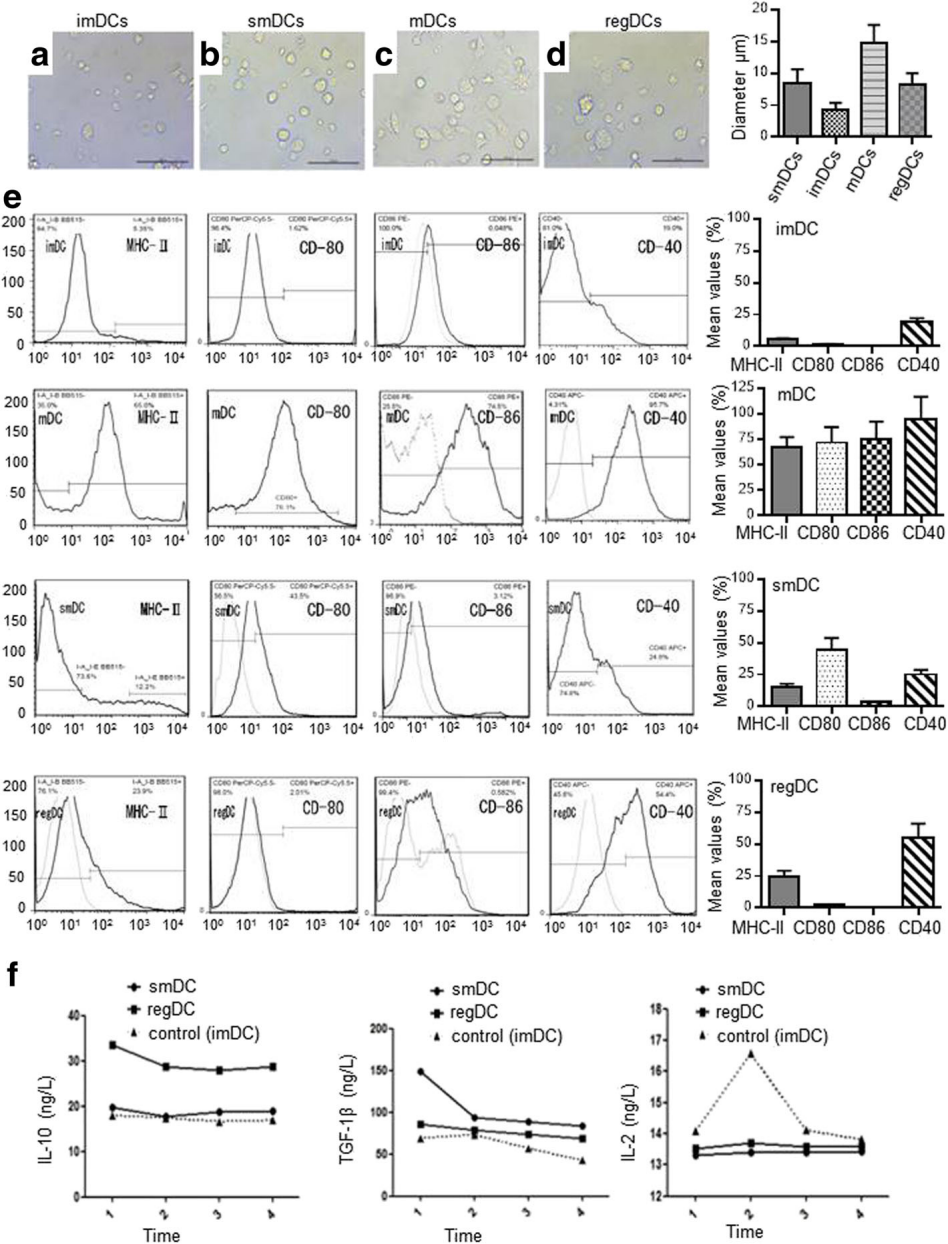
Morphological features of smDCs, mDCs, imDCs, and regDCs observed with a fully automated and inverted microscope (×200). smDCs and regDCs were either round or oval and their lengths of dendrites were between those of imDCs (**a**) and mDCs (**c**). Morphology of smDCs (**b**) and regDCs (**d**) was similar. Quantitative data about cell morphology as diameter shown in right panel. Expression of MHC-II, CD40, CD80, and CD86 on the surface of smDCs, mDCs, imDCs, and regDCs analyzed by flow cytometer. smDCs and regDCs both expressed medium levels of MHC-II, CD40, CD80, and CD86, which were higher than their corresponding levels in imDCs but lower than their corresponding levels in mDCs. (**e**). Quantitative data about the mean value of each immunophenotype shown in right panel. smDCs and regDCs also secreted high levels of inhibitory cytokines including TGF-β and IL-10, and low levels of inflammatory cytokines such as IL-2 (**f**). imDC immature dendritic cell, IL interleukin, mDC mature dendritic cell, MHC major histocompatibility complex, regDC regulatory dendritic cell, smDC semimature dendritic cell, TGF transforming growth factor

The morphology of smDCs and regDCs is similar. The cells were either round or oval and their lengths of dendrites were between those of imDCs and mDCs (Fig. 2a–d). Moreover, the types of differentiated smDCs and regDCs were between imDCs and mDCs. They both expressed medium levels of MHC-II, CD40, CD80, and CD86, which were higher than their corresponding levels in imDCs but lower than their corresponding levels in mDCs (Fig. 2e). In addition, smDCs and regDCs also secreted high levels of inhibitory cytokines including TGF-β and IL-10, and low levels of inflammatory cytokines such as IL-2 (Fig. 2f).

### Successfully induced immune tolerance in the model of hepatocyte transplantation

**Fig. 3:**
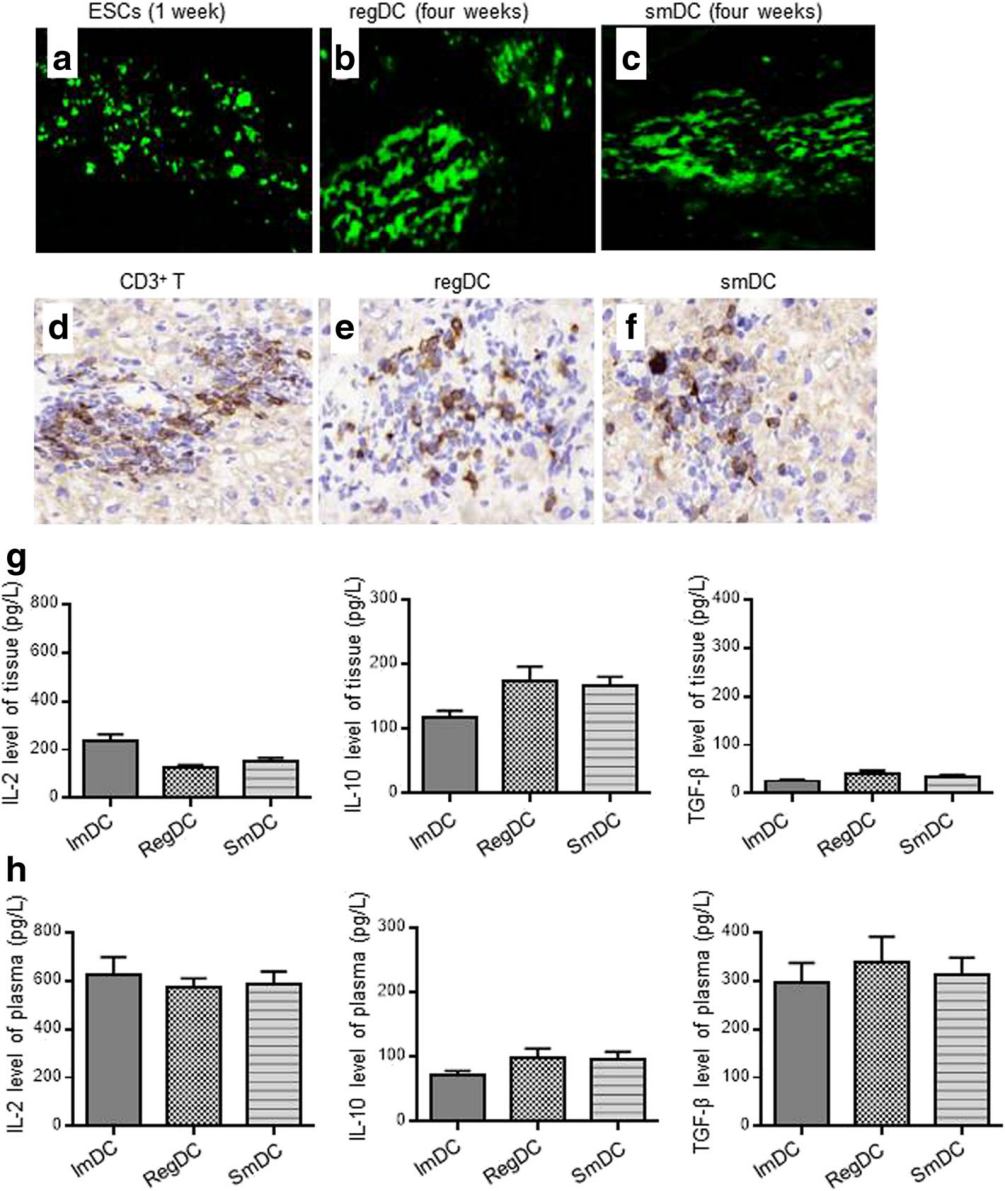
Growth of transplanted hepatocytes differentiated from the ESCs of 129 mice in the recipient and the infiltration of lymphocytes in the transplant tissues. In the control group, ESCs and their differentiated hepatocytes could survive for about 1 week (**a**). Transplanted hepatocytes differentiated from the ESCs of 129 mice grew better in the liver tissues of recipients and their survival was prolonged to about 4 weeks in the regDC (**b**) and smDC (**c**) groups. CD3^+^ T-lymphocyte infiltration in the transplant tissues was more apparent in the control group (**d**) than in the regDC (**e**) and smDC (**f**) groups. As compared with the control group (imDCs), high levels of inhibitory cytokines including TGF-β and IL-10 and low levels of inflammatory cytokines such as IL-2 were detected in transplant tissues of the smDC and regDC groups. **g** Similarly, high levels of TGF-β and IL-10 and low levels of IL-2 were also detected in the peripheral blood of the smDC and regDC groups (**h**). ESC embryonic stem cell, IL interleukin, regDC regulatory dendritic cell, smDC semimature dendritic cell, TGF transforming growth factor

**Fig. 4:**
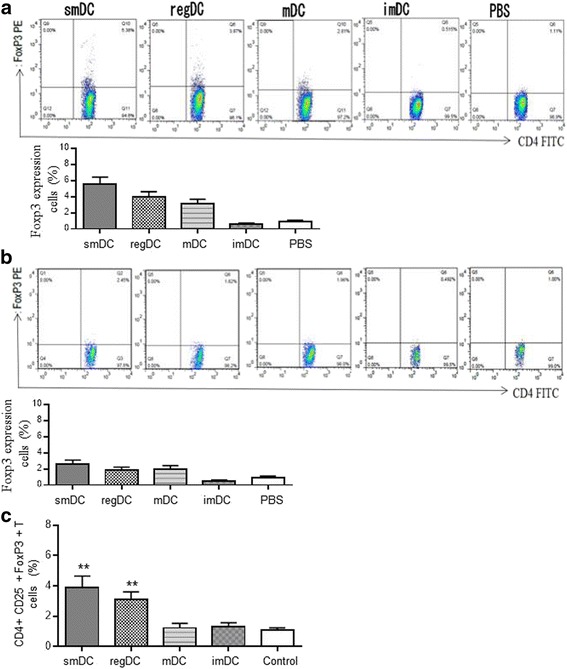
Foxp3 expression in peripheral blood CD4^+^ T cells detected by flow cytometry. **a** In the first week after transplantation, Foxp3 expression in the peripheral blood CD4^+^ T cells rose to 5.38% in the smDC group, as compared with 1.11% in the PBS group. Foxp3 expression in the peripheral blood CD4+ T cells also rose to 3.87% in the regDC group and was higher than that in the control group. **b** In the fourth week after transplantation, Foxp3 expression in peripheral blood CD4^+^ T cells of each group reduced. **c** Peripheral blood CD4^+^ T cells cultured with smDCs, regDCs, imDCs, and mDCs or PBS in vitro for 24 h, respectively. Number of CD4^+^CD25^+^FoxP3^+^ T cells detected and quantified. **P < 0.01 compared with control group. imDC immature dendritic cell, mDC mature dendritic cell, regDC regulatory dendritic cell, PBS phosphate-buffered saline, smDC semimature dendritic cell

Compared with other groups, the transplanted hepatocytes differentiated from the ESCs of 129 mice grew better in the liver tissues of recipients and their survival was prolonged to about 4 weeks. CD3^+^ T-lymphocyte infiltration in transplant tissues was reduced in the smDC and regDC groups (Fig. 3a–f). As compared with the control group, high levels of inhibitory cytokines including TGF-β and IL-10 and low levels of inflammatory cytokines such as IL-2 were detected in transplant tissues of the smDC and regDC groups. Similarly, high levels of TGF-β and IL-10 and low levels of IL-2 were also detected in the peripheral blood of the smDC and regDC groups. Moreover, it was found that Foxp3 expression in the peripheral blood CD4^+^ T cells of the smDC group rose to 5.38%, as compared with 1.11% in the PBS group. In addition, the Foxp3 expression in the peripheral blood CD4^+^ T cells of the regDC group also rose to 3.87%, which was higher than that in the control group (Fig. 4a, b). Moreover, to further show the effective regulatory activity of smDCs and regDCs, peripheral blood CD4^+^ T cells from the recipient mice were cultured with smDCs, regDCs, imDCs, and mDCs from 129 mice or PBS for 24 h in vitro, respectively. The expression of CD4^+^CD25^+^FoxP3^+^ T cells was detected. The results showed that the number of CD4^+^CD25^+^FoxP3^+^ T cells in the smDC and regDC groups was higher than those in the control group, while no significant change was found in the imDC and mDC groups compared with control group (Fig. 4c)

### ESCs showed low immunogenicity

**Fig. 5:**
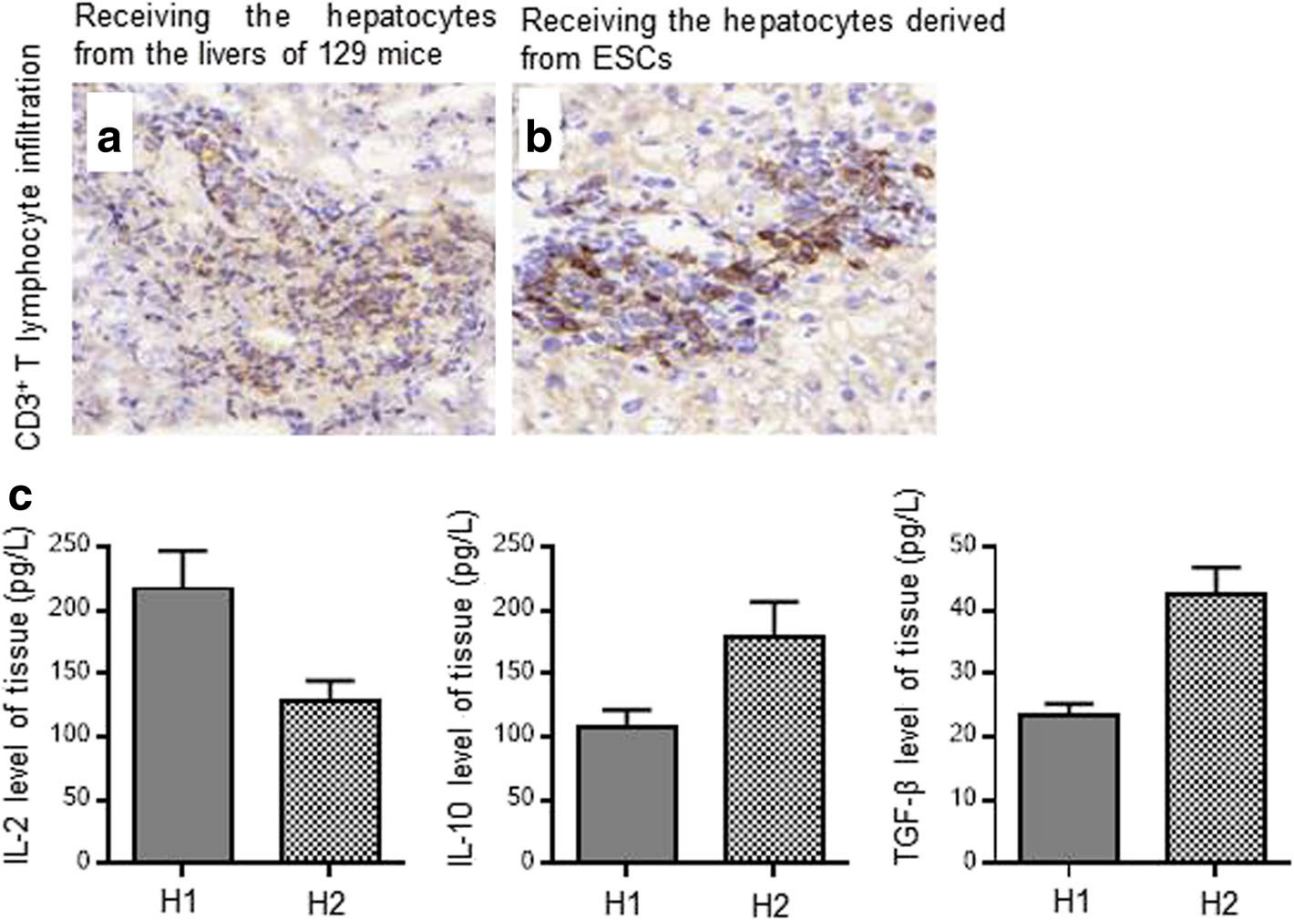
CD3^+^ T-lymphocyte infiltration and inflammatory response in transplant tissues receiving hepatocytes from livers of 129 mice and ESCs of 129 mice. Transplant tissues receiving hepatocytes from the livers of 129 mice (**a**) showed stronger CD3^+^ T-lymphocyte infiltration and a more severe inflammatory response than those receiving hepatocytes derived from ESCs (**b**). At the same time, lower levels of inflammatory cytokines such as IL-2 and higher levels of inhibitory cytokines including IL-10 and TGF-β were seen in transplant tissues receiving hepatocytes derived from ESCs compared with those receiving hepatocytes from the livers of 129 mice (**c**). ESC embryonic stem cell, H1 tissues receiving hepatocytes from the livers of 129 mice, H2 tissues receiving hepatocytes derived from ESCs, IL interleukin, TGF transforming growth factor

In each group, the transplant tissues receiving the hepatocytes from the livers of 129 mice showed stronger CD3^+^ T-lymphocyte infiltration and a more severe inflammatory response than those receiving hepatocytes derived from ESCs (Fig. 5a, b). At the same time, higher levels of inhibitory cytokines including TGF-β and IL-10 and lower levels of inflammatory cytokines such as IL-2 were seen in transplant tissues receiving hepatocytes derived from ESCs (H2) compared with those receiving the hepatocytes from the livers of 129 mice (H1) (Fig. 5c). These results indicated that ESCs showed low immunogenicity.

## Discussion

Previous studies found that as a newly discovered type of tolerogenic DCs, smDCs could help to completely alleviate autoimmune encephalomyelitis by inducing IL-10 secretion from antigen-specific CD4^+^ T cells. However, imDCs and mDCs both lack the ability to induce IL-10 secretion from CD4^+^ T cells [13, 14]. Actually, CD4^+^ T cells that can secrete IL-10 are CD4^+^CD25^+^ regulatory T cells (Tregs). Tregs are CD4^+^CD25^+^Foxp3^+^ T cells, which could be induced by smDCs expressing and secreting appropriate levels of costimulatory molecules and cytokines [15]. Tregs could affect the transduction of costimulatory signals and TCR signals, while at the same time secrete inhibitory cytokines to induce immune tolerance [16].

Moreover, as a type of tolerogenic DCs, regDCs have also been studied extensively. In in-vitro experiments, regDCs could be generated by inducing bone marrow mononuclear cells of 129 mice with rmGM-CSF, IL-10, and human TGF-β1. In mouse models of allogeneic hematopoietic stem cell transplantation, it was confirmed that, following HSCT, regDCs mitigated acute graft-versus-host disease by inducing Tregs [17, 18]. Therefore, regDCs play important roles in inducing the immune tolerance after stem cell transplantation.

Currently, ESC-derived functional cells are beneficial in terms of replacing defective cells and restoring their functions. For example, recent studies showed that human ESC-derived neural cells were beneficial to behavioral recovery in an animal model of Parkinson’s disease [19]. Our previous studies found that when ESC-derived hepatocytes were transplanted into the livers of fulminant hepatic failure mice, their life quality could be improved and their survival could be lengthened [20]. However, the risks of immune rejection remain inevitable even after the transplantation of ESC-derived cells, which have been the main reasons for the reduced therapeutic efficacy of ESC-derived cells [21]. smDCs and regDCs are both tolerogenic DCs, which play important roles in the maintenance of immune tolerance. In this study, the similarities between smDCs and regDCs in alleviating the immune injury after the transplantation of ESC-derived hepatocytes were investigated.

Firstly, a rejection model of hepatocyte transplantation was established. Subsequently, immune tolerance was successfully induced by infusing suspensions containing 129 mouse-derived smDCs into BALB/c mice in advance. It was confirmed that smDCs could alleviate the immune injury in the liver of recipients after transplantation. Foxp3 expression in the peripheral blood CD4^+^ T cells of the smDC group increased to 5.38% from 1.11%, and it was shown that smDCs maintained the immune tolerance by inducing Tregs. Moreover, it was found that the morphology of smDCs was between that of imDCs and mDCs. The smDCs expressed medium levels of MHC-II, CD40, CD80, and CD86, which were higher than their corresponding levels in imDCs but lower than their corresponding levels in mDCs. The smDCs also secreted high levels of inhibitory cytokines including TGF-β and IL-10, and low levels of inflammatory cytokines such as IL-2. In fact, an appropriate signal stimulus, including a medium level of costimulatory molecules and the secretion of related cytokines, was required for the induction of Tregs. smDCs just express an appropriate signal [22]. Accordingly, smDCs could help Treg development and maintenance by expressing medium levels of MHC-II, CD40, CD80, and CD86, and by secreting appropriate levels of cytokines. As a newly discovered type of tolerogenic DCs, smDCs could induce and augment Tregs [23].

Furthermore, the cell morphology, immunological phenotype, and cytokine secretion from regDCs were also investigated. It was found that regDCs were similar to smDCs in terms of these aspects. In addition, by inducing Tregs, regDCs and smDCs could both alleviate the immune injury in the livers of recipients after transplantation. They both express a medium level of MHC-II, CD40, CD80, and CD86. In addition, they both secrete an appropriate level of cytokines such as IL-10 and TGF-β, which are critical for the development and maintenance of Tregs [18]. Therefore, regDCs and smDCs belong to the same types of tolerogenic DCs and they can both induce the immune tolerance to transplanted cells in recipients. Such findings have provided a new idea for solving the problem of immune rejection, which usually occurs after the transplantation of stem cells and their differentiated cells.

Moreover, the transplant tissues receiving hepatocytes from the livers of 129 mice showed stronger CD3^+^ T-lymphocyte infiltration than those receiving hepatocytes derived from ESCs. Therefore, hepatocytes differentiated from ESCs are associated with low immunogenicity. According to recent findings, the immune privilege of ESCs and differentiated cells derived from ESCs might be related to the different epigenetic control of major histocompatibility and antigen processing molecules [24, 25]. It is apparent that further studies are required to elucidate those additional mechanisms.

## Conclusions

In summary, our results showed that hepatocytes differentiated from ESCs displayed low immunogenicity, but immune rejection in transplant recipients remained. Both regDCs and smDCs could alleviate the immune injury by inducing CD4^+^CD25^+^Foxp3^+^ regulatory T cells (Tregs) through the appropriate expression of MHC-II, CD40, CD80, and CD86 and the secretion of cytokines. The cells belonged to the same types of tolerogenic DCs. These findings are beneficial to promote the clinical applications of stem cell transplantation and to provide some insights into transplantation tolerance.

## Abbreviations

aFGF: Acidic fibroblast growth factor
AFP: Alpha-fetoprotein
DEX: Dexamethasone
EB: Embryoid body
ESC: Embryonic stem cell
GFP: Green fluorescent protein
GM-CSF: granulocyte–macrophage-colony stimulating factor
HGF: Hepatocyte growth factor
IL: Interleukin
imDC: Immature dendritic cell
LPS: Lipopolysaccharide
mDC: Mature dendritic cell
OSM: Oncostatin M
regDC: Regulatory dendritic cell
smDC: Semimature dendritic cell
TGF: Transforming growth factor
TNF-α: Tumor necrosis factor-alpha
